# Overexpression of 5-HT_2C_ receptors in forebrain leads to elevated anxiety and hypoactivity

**DOI:** 10.1111/j.1460-9568.2009.06831.x

**Published:** 2009-07

**Authors:** Atsuko Kimura, Paula L Stevenson, Roderick N Carter, Gavin MacColl, Karen L French, J Paul Simons, Raya Al-Shawi, Valerie Kelly, Karen E Chapman, Megan C Holmes

**Affiliations:** 1 Endocrinology Unit, Centre for Cardiovascular Science, University of Edinburgh, Queen’s Medical Research InstituteEdinburgh EH16 4TJ, Scotland; 2 Division of Medicine and Royal Free Centre for Biomedical Science, University College LondonLondon, UK

**Keywords:** 5-HT_2C_ receptor, anxiety, behaviour, transgenic mice

## Abstract

The 5-HT_2C_ receptor has been implicated in mood and eating disorders. In general, it is accepted that 5-HT_2C_ receptor agonists increase anxiety behaviours and induce hypophagia. However, pharmacological analysis of the roles of these receptors is hampered by the lack of selective ligands and the complex regulation of receptor isoforms and expression levels. Therefore, the exact role of 5-HT_2C_ receptors in mood disorders remain controversial, some suggesting agonists and others suggesting antagonists may be efficacious antidepressants, while there is general agreement that antagonists are beneficial anxiolytics. In order to test the hypothesis that increased 5-HT_2C_ receptor expression, and thus increased 5-HT_2C_ receptor signalling, is causative in mood disorders, we have undertaken a transgenic approach, directly altering the 5-HT_2C_ receptor number in the forebrain and evaluating the consequences on behaviour. Transgenic mice overexpressing 5-HT_2C_ receptors under the control of the CaMKIIα promoter (C2CR mice) have elevated 5-HT_2C_ receptor mRNA levels in cerebral cortex and limbic areas (including the hippocampus and amygdala), but normal levels in the hypothalamus, resulting in > 100% increase in the number of 5-HT_2C_ ligand binding sites in the forebrain. The C2CR mice show increased anxiety-like behaviour in the elevated plus-maze, decreased wheel-running behaviour and reduced activity in a novel environment. These behaviours were observed in the C2CR mice without stimulation by exogenous ligands. Our findings support a role for 5-HT_2C_ receptor signalling in anxiety disorders. The C2CR mouse model offers a novel and effective approach for studying disorders associated with 5-HT_2C_ receptors.

## Introduction

Central serotonergic systems modulate many behavioural and physiological functions, and thus 5-HT receptors have attracted much attention as therapeutic targets for various disorders (Barnes & Sharp, 1999). The 5-HT_2C_ receptor is one of the 14 members of the 5-HT receptor family (Martin & Humphrey, 1994), and its expression level is particularly high in the limbic system and hypothalamus where its activity is implicated in anxiety, depression, sleep disturbance, locomotor activity and feeding behaviour as well as modulation of pituitary release of ACTH and prolactin (Millan, 2005). Furthermore, mice which lack 5-HT_2C_ receptors are reported to be hyperphagic (they develop obesity in later life) and hyperactive (Tecott *et al.*, 1995), and to demonstrate less anxiety-like behaviour (Heisler *et al.*, 2007b).

Studies of 5-HT_2C_ receptor function and its pathophysiological role(s) have been difficult, largely due to a lack of selective pharmacological agents together with the complex regulation of receptor density and activity. A nonselective 5-HT receptor agonist, 1-(m-chlorophenyl)-piperazine (mCPP), which shows preferential affinity at 5-HT_2C_ receptors, has been used for inferring the role of 5-HT_2C_ receptors. In rodents, mCPP induces anxiety (Cornelio & Nunes-de-Souza, 2007; Hackler *et al.*, 2007), inhibits locomotor activity (Stiedl *et al.*, 2007) and reduces food intake (Kennett & Curzon, 1988; Hewitt *et al.*, 2002). However, careful analyses are required for such effects as mCPP also activates other 5-HT receptor subtypes to regulate activity and food intake (Nonogaki *et al.*, 2003; Dalton *et al.*, 2004; Lee *et al.*, 2004). In addition, more recently available 5-HT_2C_ receptor agonists, such as RO 60-0175, have been shown to exert a degree of action on other 5-HT_2_ receptor subtypes (Higgins *et al.*, 2001; Damjanoska *et al.*, 2003). Coupled with the lack of selective ligands, there is disagreement in the literature over whether it is the activation or inhibition of the 5-HT_2C_ receptors that causes anxiety or depressive behaviour, and hence whether it is the agonists or antagonists that could be effective antidepressants (Millan *et al.*, 2005; Dunlop *et al.*, 2006; Cremers *et al.*, 2007).

Further complexity is introduced by RNA editing of the 5-HT_2C_ receptor transcript, which potently modulates both the degree of constitutive activity of the encoded receptor and its sensitivity to ligands (Burns *et al.*, 1997). Moreover, the extent of editing is altered in different mouse strains (Englander *et al.*, 2005), following early-life stress (Bhansali *et al.*, 2007), following modulation of serotonergic neurotransmission (Gurevich *et al.*, 2002a), or in post-mortem brains from suicide victims (Gurevich *et al.*, 2002b).

Here we have tested the hypothesis that elevated 5-HT_2C_ receptor density in forebrain alters behaviour. In order to circumvent the problems associated with poor selectivity of pharmacological agents, we have undertaken a transgenic approach: we have overexpressed 5-HT_2C_ receptors in mouse forebrain and examined the resulting phenotype. The CaMKIIα–5-HT_2C_ receptor transgene promoting overexpression only expresses the unedited isoform of the receptor; consequently, the balance of the edited isoforms in our transgenic mice will be modified considerably compared with wild-type mice. We report the behavioural phenotype of transgenic mice with forebrain overexpression of 5-HT_2C_ receptors.

## Materials and methods

### Generation of transgenic mice overexpressing 5-HT_2C_ receptors in forebrain

**Fig. 1:**
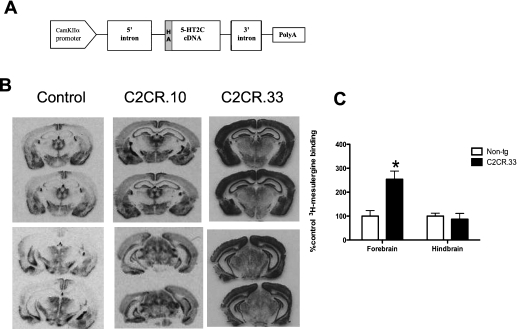
Overexpression of 5-HT_2C_ receptors in forebrain of CaMKIIα–2CR transgenic (C2CR) mice. (A) A schematic illustration of the transgene construct used for overexpressing 5-HT_2C_ receptors in forebrain; HA, haemagglutinin epitope tag. (B) Representative autoradiograph of mouse brains (control, C2CR.10 and C2CR.33) showing 5-HT_2C_ receptor mRNA levels and distribution detected by *in situ* mRNA hybridisation histochemistry. Extensive enhancement of 5-HT_2C_ receptor mRNA density was observed in the C2CR.33 mice and a more restricted increase (mainly in the dorsal hippocampus) of 5-HT_2C_ receptor mRNA level was observed in the C2CR.10 mice. (C) Elevated 5-HT_2C_ receptor binding sites in the membrane fraction isolated from C2CR.33 mouse forebrain compared with the littermate controls (*n* = 5–6; *P < 0.01). There was no difference between genotypes in the hindbrain membrane fraction. Data are mean ± SEM.

The full length (unedited) rat 5-HT_2C_ receptor cDNA was amplified by PCR from a plasmid template (pMV7-sr1cm; a gift from David Julius, San Francisco, USA). An influenza haemagglutinin (HA) epitope tag was added at the N-terminus of the receptor by subcloning a double-stranded oligonucleotide encoding the HA tag at a *Kpn*I site generated by PCR at the translation start of the 5-HT_2C_ receptor cDNA. Receptor function is not altered by the N-terminal addition of this tag (Hurley *et al.*, 1999). The HA–5-HT_2C_ receptor cDNA was then subcloned into the *EcoRV* site of pNN265 (Mayford *et al.*, 1996). Transient transfection of this plasmid into Cos7 cells conferred binding of the 5-HT_2C_ receptor ligand ^3^H-mesulergine (data not shown). A *Not1* fragment including the HA–5-HT_2C_ receptor (HA-2CR) cDNA was then inserted into the NotI site of pMM403, encoding the mouse calcium–calmodulin-dependent protein kinase (CaMK) IIα promoter (Mayford *et al.*, 1996), to create the transgene construct CaMKIIα–HA-2CR (Fig. 1A). All constructs were verified by DNA sequencing. Transgenic mice were generated by pronuclear injection of the 11-kb CaMKIIα–HA-2CR minigene construct into C57BL/6J × CBA embryos. Four founder mice (designated C2CR) were obtained, all of which transmitted the transgene. Two C2CR lines (C2CR.10 and C2CR.33), which demonstrated similar traits, were selected for further characterisation. Mice were backcrossed to C57BL/6 and they were genotyped by PCR using primers corresponding to the HA tag (forward: 5′-ATGTACCCATACGATGTTCCAGATTACGCT-3′) and complementary to the 5-HT_2C_ receptor cDNA (reverse: 5′-CAGAGGTGCATGGACGC-3′), giving a product of 420 bp. In some experiments, genotypes were confirmed by Southern blot analysis of the *EcoRV* digested genomic DNA from C2CR mice, probed with a ^32^P-labelled DNA fragment comprising the 2.7-kb *BamH1* fragment of the transgene. Southern blotting was also used to confirm a single integration site of the transgene in C2CR.10 and C2CR.33 mice, consistent with the observed ∼50% transmission rate for the transgene. Experimental mice were F3–F5.

### Animals

Animals were given standard chow and water *ad libitum*, lights were on between 07:00 h and 19:00 h, and all studies were performed to the highest standards of humane animal care under the aegis of the United Kingdom Animals Scientific Procedures Act, 1986. In all experiments, nontransgenic littermates of the corresponding C2CR mice served as controls. For *in vitro* experiments, male mice (30–35 g) were killed by cervical dislocation and whole brain was immediately removed and frozen on dry ice.

### Detection of 5-HT_2C_ receptor mRNA by *in situ* hybridisation

*In situ* mRNA hybridisation was performed as described previously (Holmes *et al.*, 1995, 1997). In brief, brain sections (15 μm) were thaw-mounted on silane-coated slides and post-fixed in 4% paraformaldehyde in a 0.1 m phosphate buffer followed by three washes in PBS, then dehydrated in increasing concentrations of ethanol. Sections were incubated in a prehybridisation buffer (deionised formamide, 50%; NaCl, 600 mm; Tris–HCl, pH 7.5, 10 mm; EDTA, 1 mm; 1 × Denhardt’s solution; salmon sperm DNA, 0.5 mg/mL; and yeast tRNA, 0.1 mg/mL) for 2 h at 50°C. [^35^S]-UTP-labelled probe for detecting endogenous 5-HT_2C_ receptors (complementary to the 5′-UTR, absent from the transgene) was prepared as described previously (Holmes *et al.*, 1997). Transgene expression was detected with a [^35^S]-UTP labelled HA-2CR riboprobe corresponding to the HA tag and first 400 bp following the translation start of the rat 5-HT_2C_ receptor cDNA. This riboprobe also hybridises to the endogenous 5-HT_2C_ receptor mRNA. [^35^S]-UTP-labelled probes (10^7^ cpm/mL) were hybridised to brain sections and stringent washes were carried out as described previously (Holmes *et al.*, 1995). Brain sections were exposed to a Kodak® BioMax MR film (Sigma-Aldrich, Poole, UK) for 3–8 days, and optical density for the area of interest was measured using an image analysis software MCID-M4, ver 3.0, rev.1.5 (Interfocus Imaging Ltd, Cambridge, UK). Data are expressed as percentages of values in control animals.

### Radioligand binding assay for 5-HT_2C_ receptors

Radioligand binding assays for 5-HT_2C_ receptors were carried out as described previously (Marazziti *et al.*, 1999), with slight modifications. In brief, forebrains or hindbrains from C2CR and control mice (frozen on dry ice and stored at −80°C until use) were homogenised in a buffer containing 0.32 m sucrose, 50 mm Tris–HCl (pH 7.4), 0.1% ascorbate and protease inhibitors (Mini Protease inhibitor cocktail tablets, Roche Applied Science, West Sussex, UK), then centrifuged at 300 ***g*** for 5 min at 4°C. The supernatant was removed and centrifuged at 50 000 ***g*** for 15 min at 4°C. The resulting pellet was washed and resuspended in an ice-cold buffer containing 50 mm Tris–HCl (pH 7.4), 0.1% ascorbate and protease inhibitors, for use in the assay. Total binding in membranes (0.6 mg/mL protein) was determined in the presence of 10 nm^3^H-mesulergine (specific activity: 2.85 Tbq/mm; GEHealthcare, Amersham, UK) and 100 nm spiperone (Sigma), for blocking ^3^H-mesulergine binding to 5-HT_2A_ receptors, while nonspecific binding was determined with an addition of 1 mm 5-HT (Sigma). The membranes were incubated with the aforementioned ligands, as appropriate, for 30 min at 37°C. Membrane incubation was terminated by rapid filtration using a Combi cell harvester (Skatron Instruments, Lier, Norway), filters were washed and dried, and then the radioactivity remaining on the filters was quantified using a liquid scintillation counter (Packard tri-carb 2100TR, Packard Instruments, Berks, UK).

### Measurement of activity in wheel-running test

Male C2CR and control mice were individually housed in wheel cages (wheel diameter of 23.5 cm) and wheel revolutions were counted using Clocklab acquisition and analysis computer software (ActiMetrics, IL, USA) throughout the whole experimental period. Average daily revolutions were calculated over a 7-day period, following a 5-day acclimatisation period. Activity of mice during the first 1–2 h from the start of dark phase (i.e. 19:00–21:00 h) was recorded to determine the phenotypic and drug-induced effects.

### Behavioural tests

For the elevated plus-maze (EPM) and open-field tests, male C2CR and control mice were individually caged 48 h prior to the tests, then moved from the holding room to the behaviour room 2 h prior to the tests for acclimatisation. Each mouse undertook up to three behavioural tests in random order with an interval of 1 week between tests, except when they were scheduled for the EPM test. The EPM test was always carried out first as the behaviour in this test was influenced by the pre-exposure to other behavioural tests (data not shown). Mice and drug treatments used in the behavioural tests were randomised in a double-blind manner.

### Open-field test

The open-field test was performed as described previously (Holmes *et al.*, 2006). In brief, the open-field arena consisted of a square box (50 × 50 cm, 25 cm high, grey Perspex) divided into 5 × 5 grids, with the central nine squares defined as the inner zone and the rest as the outer zone. A male C2CR or control mouse was placed in a corner of the box and its activity was monitored and recorded for 5 min using a computer tracking system, Limelight (ActiMetrics, IL, USA). The number of crossings into the inner zone, time spent in the inner zone and distance travelled within the inner zone were analysed.

### EPM test

The EPM test was performed as described previously (Holmes *et al.*, 2006). In brief, the maze consisted of a perspex platform in a shape of a plus sign, raised 1 m above ground. One opposing pair of arms was enclosed by high walls (closed arms) and the other opposing arms were exposed (open arms). A male C2CR or control mouse was placed in the centre of the plus-maze, where all the arms met, and its behaviour was monitored and recorded immediately thereafter for 5 min using a computer tracking system (Limelight). The number of open-arm entries, time spent in the open arms and distance travelled within the open arms were analysed. Ethological parameters such as stretch–attend (stretching out from enclosed arms over the side of the open arm), rearing, grooming, immobility and faeces were scored manually.

### Tail-suspension test

The tail-suspension test is used to determine depressive-like characteristics, indicated by the time spent immobile during the test. A male C2CR or control mouse was suspended above the table top using an adhesive tape placed ∼0.5 cm from the tip of its tail. Total duration of immobility was measured over a 6-min period. Mice were considered to be immobile only when they hung down passively and were completely motionless. Mice were scored every 5 s as either S (struggling) or H (hanging). Hanging-to-struggling ratios and the percentage time spent hanging were also calculated.

### Drug treatment

In order to elucidate the role of 5-HT_2C_ receptors in the behavioural tests, mice were injected with the following drugs: 5-HT receptor agonists mCPP (0.3–3 mg/kg; Sigma) and (S)-2-(chloro-5-fluoro-indol- l-yl)-1-methylethylamine fumarate (RO 60-0175; 3–5 mg/kg; Tocris Bioscience, Bristol, UK); the 5-HT_2C_ receptor antagonist 6-chloro-5-methyl-1-[6-(2-methylpiridin-3-yloxy)pyridin-3-yl carbamoyl] indoline (SB242084; 3 mg/kg; Sigma), the 5-HT_2C_ receptor inverse agonist 5-methyl-1-(3-pyridylcarbamoyl)-1,2,3,5-tetrahydropyrrolo[2,3-f] indole hydrochloride (SB206553; 1–5 mg/kg; Tocris Bioscience) or vehicle (10% ethanol in saline for all drugs except mCPP and RO 60-0175, which were dissolved in saline). The drugs were administered i.p. 30 min prior to testing or (for wheel-running behaviour) 30 min prior to entering the dark phase (i.e. injected at 18:30 h).

### Measurement of plasma corticosterone

For the measurement of basal plasma corticosterone, blood samples were taken between 07:00 and 08:00 h, by tail venesection within < 30 s after disturbing the mouse in its home cage. For peak stress plasma samples, trunk blood was collected 10 min after a mouse was placed in a Perspex restraint tube. Blood was collected in EDTA-coated Microvette tubes (Sarstedt, Numbrecht, Germany) and centrifuged, and plasma was stored at −20°C until corticosterone determination by radioimmunoassay as described previously (Holmes *et al.*, 1997).

### Statistical analysis

The genotypic effect was analysed using a Student’s *t*-test. For the behavioural studies, one- or two-way anova was used as appropriate, followed by a Dunnett’s *post hoc* test. Data are expressed as the mean ± SEM.

## Results

### Generation of transgenic mice overexpressing 5-HT_2C_ receptors in forebrain

Four lines of C2CR transgenic mice were generated following pronuclear microinjection of a CaMKIIα–HA-2CR minigene (Fig. 1A), in which the CaMKIIα promoter (forebrain-specific) drives the expression of unedited 5-HT_2C_ receptors. In order to facilitate transgene detection, an HA epitope tag was added to the N-terminus of the receptor, a modification that does not affect receptor function (Hurley *et al.*, 1999).

All C2CR mouse lines were viable and healthy with normal body weight and displayed no gross abnormalities (data not shown). The average litter size and gender ratio were unaffected by the transgene integration. Southern blotting confirmed the transgene integration at a single site (data not shown). Based on the expression pattern of the transgene in the forebrain, two lines were selected for further characterisation: C2CR.33, in which 5-HT_2C_ receptor mRNA levels are high and widely distributed throughout the forebrain including the cerebral cortex, hippocampus and amygdala (Fig. 1B and Table 1), and C2CR.10, in which the distribution of 5-HT_2C_ receptor mRNA is broadly similar to C2CR.33 but the level of overexpression is lower than in C2CR.33 (Fig. 1B and Table 1). The level of endogenous 5-HT_2C_ receptor mRNA expression in both C2CR mouse lines was unaltered compared with the controls (data not shown). ^3^H-mesulergine binding to forebrain membranes from C2CR.33 mice was over two-fold greater than the controls (Fig. 1C), indicating the increased 5-HT_2C_ receptor mRNA level was translated into higher receptor levels at the cell surface. The level of ^3^H-mesulergine binding sites was not altered in the hindbrain membranes where the transgene is not expressed (Fig. 1C). Initial phenotypic characterisation was carried out in both C2CR mouse lines and similar data were obtained despite the differences in the transgene expression levels.

**Table 1:** 5-HT_2C_ receptor mRNA expression in C2CR mouse brain

	5-HT_2C_ receptor mRNA expression (% of control mice)	
Brain area	C2CR.10	C2CR.33
Dorsal hippocampus		
Dentate gyrus	20476 ± 1992**	3146 ± 369**
CA1	709 ± 80**	1688 ± 210**
CA3	633 ± 67**	2373 ± 319**
Cerebral Cortex	322 ± 84*	380 ± 42*
Amygdala	101 ± 9	250 ± 29*
Choroid plexus	105 ± 8	87 ± 9

The percentage change in 5-HT_2C_ receptor mRNA expression in the C2CR mouse brains (C2CR.10 and C2CR.33 mice) relative to the corresponding control mice. The expression levels were determined by *in situ* hybridisation of brain sections followed by densitometric analysis of autoradiographs exposed to the hybridised brain sections. Data are mean ± SEM; *n* = 6; *P < 0.05, **P < 0.001 compared with the control mouse brain.

### Reduced locomotor activity in C2CR mice

**Fig. 2:**
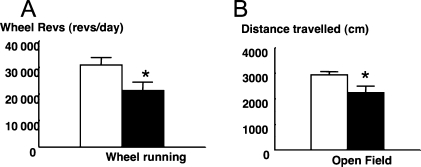
C2CR mice showed decreased activity. (A) Voluntary wheel-running activity: mice were individually placed in a cage with free access to a running wheel connected to a system for recording the number of wheel revolutions (a measurement of activity levels). The average number of wheel revolutions (over 7 days) generated by the C2CR mice was significantly lower than for the controls. (B) Open-field (novel environment) test: mice were individually placed in an open-field arena and their movement monitored for 5 min. Total distance travelled within the open-field arena in the C2CR mice was significantly less than for the controls. White bar, control mice; black bar, C2CR mice. Data are mean ± SEM; *n* = 6–8, *P < 0.05 genotypic comparison.

Daily wheel-running activity in the C2CR mice was significantly lower than in the control mice (Fig. 2A). Also, the distance travelled by the C2CR mice in a novel environment (open field) was significantly less than the controls (Fig. 2B). These results suggest that an increased forebrain 5-HT_2C_ receptor expression reduces locomotor activity.

### C2CR mice showed increased anxiety-like behaviour

**Fig. 3:**
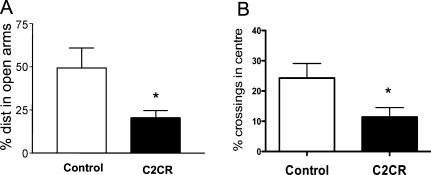
C2CR mice showed increased anxiety-like behaviour. (A) EPM test: mice were placed individually in the centre of the EPM and their movement monitored for 5 min. The % distance travelled in the more anxiogenic open arms in the C2CR mice (black bar) was significantly lower than the controls (white bar). (B) Open-field test: mice were placed individually in one corner of the open-field arena and their movement monitored for 5 min. The ratio of central to total crossings in the open-field arena was used for quantifying the anxiety component in this test. The percentage crossing into the more anxiogenic central zone in the C2CR mice (black bar) was lower than for the controls (white bar). Data are mean ± SEM; *n* = 8–11, *P < 0.05 genotypic comparison.

The total distance travelled in the EPM was similar between genotypes for both C2CR lines, indicating that this paradigm was unable to detect the general hypolocomotor effects of the 5-HT_2C_ receptor overexpression (data not shown). However, the distance travelled within the open arms by the C2CR mice was significantly less than by the controls (Fig. 3A), indicating an increased anxiety-like behaviour in the C2CR mice. Ethologically-derived behavioural measures in this test showed no significant difference between genotypes for the number of stretch–attends (out of the closed arms over the side of the open arms), rears, faeces or bouts of grooming or immobility (data not shown). However, the time spent investigating the open arms (stretch–attend), indicative of a risk-assessment strategy, was significantly greater in the C2CR mice (19.8 ± 5.0 s, *n* = 10) than in the controls (8.3 ± 0.9 s, *n* = 9; *P* = 0.04). Also, there was a tendency for the C2CR mice to spend more time immobile during the test than did the controls (C2CR mice, 32.9 ± 16.5 s, *n* = 10; controls, 11.9 ± 7.1 s, *n* = 9; NS). Consistent with the increased anxiety, in the open-field test the C2CR mice showed fewer crossings into the central area of the open-field arena as a percentage of total crossings than did the control mice (Fig. 3B).

In the tail-suspension test, there was no difference between the genotypes for the depressive-like behaviour, indicated by the percentage time spent hanging immobile (controls, 50.36 ± 2.64%, *n* = 27; C2CR mice, 54.72 ± 2.21%, *n* = 20; NS), suggesting that overexpression of 5-HT_2C_ receptors in the forebrain does not induce a depressive-like behaviour.

The increased anxiety-like behaviour was not accompanied by (or could not be ascribed to) activation of the hypothalamic–pituitary–adrenal (HPA) axis as plasma corticosterone levels were the same in the C2CR and control mice, at both basal and stressed states (Table 2). Furthermore, chronic alteration in the HPA axis activity is unlikely as adrenal weights were also identical in the two genotypes (Table 2), suggesting that there was no major modification in the long-term corticosterone secretion as a result of increased level of forebrain 5-HT_2C_ receptors.

**Table 2:** Basal and peak stress plasma corticosterone levels and adrenal weights in C2CR mice

	Control	C2CR
Plasma corticosterone (nm)		
Basal	58.3 ± 18.1	63.2 ± 8.1
Stress	257.0 ± 89.4*	254.3 ± 33.6*
Adrenal weight (mg)	0.6 ± 0.04	0.6 ± 0.01

Plasma corticosterone levels were determined in the C2CR and control mice at two states: unstressed (basal) and stressed (after 10 min restraint). Stress was applied using a restraint tube and the blood samples were collected once between 07:00 h and 08:00 h. Stress increased plasma corticosterone levels in both control and C2CR mice, but there was no genotypic difference. Adrenal weights were almost identical in the control and C2CR mice. Data are mean ± SEM; *n* = 6; *P < 0.01 compared with the respective basal levels.

### Effect of 5-HT_2C_ receptor ligands on wheel-running behaviour in C2CR mice

The hypolocomotor effects of transgene expression could be due to an elevated forebrain 5-HT_2C_ receptor number, an increased ligand sensitivity of transgene-encoded 5-HT_2C_ receptor (the unedited isoform has a higher constitutive activity and an increased ligand sensitivity *in vitro*) or a combination of the two. In order to address this question, 5-HT_2C_ receptor ligands or vehicle were administered to the C2CR mice (i.p.) and effects on locomotor activity in wheel running were measured for 2 h following the onset of the dark phase (i.e. during the period 19:00–21:00 h). The C2CR mice showed clearly a reduced activity compared with the controls in all conditions: basal (no injection), or following saline or mCPP (3 mg/kg) administration (*F*_1.24_ = 14.27, *P* < 0.001; Table 3). Administration of mCPP decreased the wheel-running activity compared with the saline injection alone by 64.4 ± 19.2% in the controls and by 79.6 ± 9.7% in the C2CR mice (*n* = 6). The effect of mCPP was highly significant (*F*_2.24_=29.0, *P* = 0.0001), but there was no interaction of drug and genotype. A lower dose of mCPP (0.3 mg/kg) had no effect in either genotype compared with the saline injection (data not shown).

**Table 3:** Effects of 5-HT_2C_ receptor antagonist and inverse agonist on wheel-running behaviour in C2CR mice

Treatment and time of day	Control (revolutions)	C2CR (revolutions)
Basal		
19:00–20:00 h	5038 ± 318	3228 ± 912*
20:00–21:00 h	4405 ± 657	2379 ± 842
Vehicle		
19:00–20:00 h	4462 ± 420	1523 ± 1050*
20:00–21:00 h	3791 ± 971	2603 ± 844
Antagonist SB242084 (3 mg/kg)		
19:00–20:00 h	3751 ± 916	2093 ± 784*
20:00–21:00 h	3445 ± 1054	1671 ± 772
Inverse agonist SB206553 (1 mg/kg)		
19:00–20:00 h	3149 ± 1175	1907 ± 681
20:00–21:00 h	3794 ± 913	1254 ± 570
Inverse agonist SB206553 (5 mg/kg)		
19:00–20:00 h	1401 ± 440^†^	351 ± 325*^,†^
20:00–21:00 h	2074 ± 713	1097 ± 687

The number of wheel revolutions generated by the control and C2CR mice in the 2 h subsequent to the lights being switched off were counted in untreated mice (basal) and treated mice: 30 min following the administration of vehicle, antagonist (SB242084; 3 mg/kg) or inverse agonist (SB206553; 1 or 5 mg/kg). While the C2CR mice generally carried out less wheel running than the control mice (*F*_1.50_ = 13.0, *P* < 0.001), there were differences between the treatment groups (*F*_4.50_=4.9, *P* < 0.01). However, the effect of the drug treatment was similar across the genotypes (i.e. no interaction). Data are mean ± SEM; *n* = 6; *P < 0.05 compared with the respective control mice; ^†^*P* < 0.05 compared with the respective saline group.

In order to further examine whether the reduced wheel-running activity in the C2CR mice is due to higher sensitivity of the receptors to endogenous 5-HT or increased constitutive receptor activity, a selective antagonist (SB242084) or an inverse agonist (SB206553) was injected 30 min prior to the onset of the dark phase. SB242084, at doses previously shown to antagonise ligand-activated 5-HT_2C_ receptors in mouse (Stiedl *et al.*, 2007), failed to affect the wheel-running behaviour in both control and C2CR mice (Table 3). This suggests the hypolocomotion in the C2CR mice may be due to increased constitutive activity of overexpressed 5-HT_2C_ receptors rather than an increased ligand activation of 5-HT_2C_ receptors by endogenous 5-HT. Administration of SB206553 was expected to antagonise the constitutive activity of 5-HT_2C_ receptors and hence increase locomotion, particularly in the C2CR mice. However, SB206553 at higher doses (5 mg/kg, i.p.) paradoxically decreased locomotion in both control and C2CR mice (Table 3). The specificity of SB206553 for 5-HT_2C_ receptors was tested by administration of this drug to 5-HT_2C_ receptor-deficient mice, in which it also reduced activity (M. C. Holmes, P. L. Stevenson and R. N. Carter, unpublished data). This demonstrates that in mouse, contrary to rat, SB206553 inhibits locomotion through a mechanism distinct from the 5-HT_2C_ receptor signalling. Therefore, SB206553 was unable to elucidate the constitutive activity of the 5-HT_2C_ receptors in the C2CR mice.

### Effect of 5-HT_2C_ receptor agonists on locomotor behaviour in the open-field test

**Fig. 4:**
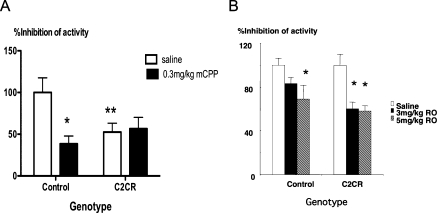
Effect of 5-HT_2C_ receptor agonists on locomotor activity in 2CR mice in a novel environment. (A) Distance travelled in the open-field arena was determined in control and C2CR mice following saline or mCPP (0.3 mg/kg) injection (i.p.). The distance travelled by control mice was significantly decreased following the mCPP treatment. However, mCPP had no effect on the distance travelled in C2CR mice compared with saline treatment. (B) The effect of RO 60-0175 on the inhibition of activity in the open-field test was compared with the effect of saline alone in the control or C2CR mice. Mice were administered RO 60-0175 (3 mg/kg, black bar; 5 mg/kg, striped bar) or vehicle (open bar) i.p., 30 min prior to testing activity in the open-field arena for 5 min. The higher dose of RO 60-0175 (5 mg/kg) reduced the activity of both control and C2CR mice, whereas the lower dose (3 mg/kg) was only efficacious in the C2CR mice. Data are mean ± SEM; *n* = 9–11; *P < 0.05 compared with saline in respective genotypes; **P < 0.05 compared to saline injection in control mice.

In order to test the acute effect of 5-HT_2C_ receptor agonists on movement in a novel environment, locomotion was assessed in the open-field test. This test evaluates both locomotor activity and anxiety-like behaviour. C2CR and control mice were injected with saline or mCPP, 30 min prior to observation of activity in the open field. A two-way anova revealed that there was a significant effect of drug treatment on activity (*F*_1.36_=4.81, *P* = 0.03) but no effect of genotype; however, there was a significant interaction of the two variables (*F*_1.36_=6.28, *P* = 0.017), indicating that the effect of mCPP was dependent on genotype. When the C2CR and control mice were analysed independently, compared with the saline treatment, mCPP (0.3 mg/kg) significantly reduced both the total distance travelled (Fig. 4A) and the total number of crossings (data not shown) in the control mice whereas it had no effect upon the activity of the C2CR mice (Fig. 4A). In contrast, when the effect of RO 60-0175 (a more selective agonist) was examined under the same conditions, RO 60-0175 inhibited locomotion in both control and C2CR mice at 5 mg/kg, while only the C2CR mice were affected by the lower dose (3 mg/kg; Fig. 4B).

## Discussion

We have tested the hypothesis that increased 5-HT_2C_ receptor signalling may be causative of anxiety and reduced activity, and provide a proof-of-principle that increased 5-HT_2C_ receptor levels in the forebrain alter behaviour. We have generated transgenic mice whose forebrain 5-HT_2C_ receptors are overexpressed. The transgene expression in our transgenic (C2CR) mice was driven by the well-characterised CaMKIIα promoter which increased the 5-HT_2C_ receptor levels in the limbic regions and cerebral cortex, a pattern of expression that is similar (though not identical) to the endogenous 5-HT_2C_ receptor expression (Palacios *et al.*, 1990; Holmes *et al.*, 1995). The C2CR mice demonstrated that forebrain 5-HT_2C_ receptor overexpression results in hypolocomotion and increased anxiety-like behaviour.

The two C2CR mouse lines, one with high and the other with modest levels of 5-HT_2C_ receptor overexpression in the forebrain, showed a consistent phenotype, suggesting that the observed phenotype is as a result of the 5-HT_2C_ receptor overexpression and is not due to a disruption of an unknown gene by a random chromosomal insertion of the transgene. There were no differences in the body weight or basal and stress-induced plasma corticosterone levels between the C2CR mice and their littermate controls. These results are consistent with the lack of transgene expression of 5-HT_2C_ receptors in the hypothalamic areas, which is involved in regulation of feeding or indeed regulation of corticotropin-releasing factor (Heisler *et al.*, 2007a; Lam *et al.*, 2008) and, hence, involved in corticosterone production. Therefore, this eliminates the possibility that the elevated anxiety-like behaviour in the C2CR mice results from a hypersensitive HPA axis.

Our data show that elevated 5-HT_2C_ receptors levels in the C2CR mice generate anxiety-like behaviour in the absence of exogenous ligand administration, suggesting that these receptors are already activated by the endogenous ligand and/or they are constitutively active. Our observation that the C2CR mice demonstrate greater anxiety-like behaviour in the EPM test and in the open-field test is consistent with previous studies demonstrating that 5-HT_2C_ receptor agonists (i.e. increased 5-HT_2C_ receptor signalling) elevate anxiety, while the antagonists are anxiolytic (Kennett *et al.*, 1989; Jenck *et al.*, 1998; Alves *et al.*, 2004). Indeed, antagonists have shown not only anxiolytic properties but also antidepressant properties (Millan *et al.*, 2005; Dekeyne *et al.*, 2008). However, the antidepressant properties of agomelatine (Millan *et al.*, 2005) may in part be attributed to its melatonin agonist activity. Conversely, 5-HT_2C_ receptor agonists, rather than antagonists, have also been demonstrated to be efficacious antidepressants (Dunlop *et al.*, 2006). Interestingly, the C2CR mice do not display depression-like behaviour in the tail-suspension test, suggesting that increased signalling through 5-HT_2C_ receptors does not lead to depression. However, evaluation of more tests for depression are warranted (prior to and following antidepressant treatment) before a depressive-like state can be ruled out in these transgenic mice.

The C2CR mice were hypoactive in two different behavioural paradigms: voluntary activity measured in wheel running and activity assessed in a novel environment with a component of stress (open field). Hypoactivity is also observed in response to 5-HT_2C_ receptor agonists (Stiedl *et al.*, 2007) underpinned by 5-HT_2C_ receptor regulation of dopamine release (Di Matteo *et al.*, 1999, 2004; De Deurwaerdere *et al.*, 2004). 5-HT_2C_ receptor agonists inhibit mesocortical dopaminergic pathways via recruitment of GABA interneurones, while antagonists exert a facilitatory influence (Di Giovanni *et al.*, 1999; Gobert *et al.*, 2000; Invernizzi *et al.*, 2007). Indeed, preliminary evidence of altered dopamine signalling in C2CR transgenic mice was obtained from an increased ratio of DA : DA metabolite content in the cerebral cortex (P.L. Stevenson and M.C. Holmes, unpublished data). Taken together, the hypoactivity observed in the C2CR mice is consistent with increased 5-HT_2C_ receptor signalling in the frontal cortex, resulting in inhibition of dopamine release.

The role of 5-HT_2C_ receptors in locomotor behaviour was further investigated using pharmacological agents. 5-HT_2C_ receptor agonists, mCPP and RO 60-0175, decreased activity of mice in both the novel environment of the open field and the voluntary wheel running. Intriguingly, the level of agonist-induced inhibition of activity did not dramatically differ between the genotypes regardless of the distinct receptor expression levels. Only the submaximal dose of RO 60-0175 distinguished between the genotypes; the hypoactivity induced by RO 60-0175 in the C2CR mice was greater than in the controls, the effect reflecting the higher 5-HT_2C_ receptor number in the C2CR mice.

Interestingly, the vehicle injection alone caused hypoactivity in all mice in both the open-field and wheel-running tests. This was independent of the acclimatisation period as mice in wheel-running cages showed highly reproducible running behaviour over 1 or 2 weeks in the cages yet, as soon as they were removed from the cage for injection, the stress inhibited activity patterns following this intervention. In some cases the transgenic mice responded to this stress of the saline injection so severely that no further inhibition of activity could be observed with the agonist (presumably a floor effect due to maximal 5-HT activation of receptors in response to stress). However, overall there was not a statistically significant difference between the response of the transgenic mice and the control mice. The acute nature of these effects (within 30 min) suggests that the effect was not mediated by increased corticosterone effects (which would require genomic action, and > 40 min delay), but was a direct effect on 5-HT release, or indeed other neurotransmitter pathways, in response to the stress. The failure of the selective 5-HT_2C_ receptor antagonist, SB242084, to elevate activity levels in doses previously shown to be inhibitory *in vivo* (Kennett *et al.*, 1997; Hayashi *et al.*, 2004; Nonogaki *et al.*, 2007) suggests that the hypoactivity was due not to ligand activation of 5-HT_2C_ receptors (ie. there is a low level of endogenous 5-HT release under basal conditions) but to constitutive activity of the 5-HT_2C_ receptors in the C2CR mice. However, administration of SB206553, a drug previously shown to be an inverse agonist at 5-HT_2C_ receptors (SB206553) in rat *in vivo* (De Deurwaerdere *et al.*, 2004; Berg *et al.*, 2006) and on human 5-HT_2C_ receptors in cell culture, did not restore hypoactivity; it instead decreased activity of mice in both wheel-running and open-field tests. This effect was presumed to be independent of the drug action on 5-HT_2C_ receptors as similar effects, to the same degree, were also observed in 5-HT_2C_ receptor-deficient mice (M. C. Holmes, P.L. Stevenson and R. N. Carter, unpublished data). Hence, at present we are unable to determine pharmacologically whether the overt behavioural phenotype observed in the C2CR mice is as a result of increased constitutive activity or enhanced sensitivity to the endogenous ligand of 5-HT_2C_ receptors. However, it is likely that the answer is a combination of the two as the transgene only expresses the unedited form of the receptor, which has been shown to be both constitutively active and more sensitive to ligand than the edited forms *in vitro* (Niswender *et al.*, 1999). Interestingly, a mouse model expressing only the unedited (INI) isoform, but at normal levels, has recently been produced (Kawahara *et al.*, 2008). Such mice have no overt metabolic phenotype which would be indicative of increased signalling through this receptor, but the consequences of this genetic manipulation on anxiety and depressive-like behaviours has yet to be determined.

In conclusion, our animal model of unedited 5-HT_2C_ receptor overexpression in forebrain provides the first evidence that an overexpression of 5-HT_2C_ receptors *in vivo* elicits a phenotype of elevated anxiety-like behaviour and hypolocomotion, consistent with an increased 5-HT_2C_ receptor signalling. This model offers an alternative method to studying 5-HT_2C_ receptors using solely pharmacological agents, which at present lack selective ligands. It now becomes important to determine the molecular and cellular consequences of 5-HT_2C_ receptor overexpression and altered profile of editing, which will aid our further understanding of the function of 5-HT_2C_ receptors and their role in mood disorders.

## Abbreviations

C2CR mice: transgenic mice overexpressing 5-HT_2C_ receptors under the control of the CaMKIIα promoter
CaMK: calcium–calmodulin-dependent protein kinase
EPM: elevated plus-maze
HA: haemagglutinin
HA-2CR: HA–5-HT_2C_ receptor
HPA: hypothalamic–pituitary–adrenal
mCPP: 1-(m-chlorophenyl)-piperazine
RO 60-0175: (S)-2-(chloro-5-fluoro-indol- l-yl)-1-methylethylamine fumarate
SB206553: 5-methyl-1-(3-pyridylcarbamoyl)-1,2,3,5-tetrahydropyrrolo[2,3-f] indole hydrochloride
SB242084: 6-chloro-5-methyl-1-[6-(2-methylpiridin-3-yloxy)pyridin-3-yl carbamoyl] indoline
